# A decision support system based on radiomics and machine learning to predict the risk of malignancy of ovarian masses from transvaginal ultrasonography and serum CA-125

**DOI:** 10.1186/s41747-021-00226-0

**Published:** 2021-07-26

**Authors:** Valentina Chiappa, Matteo Interlenghi, Giorgio Bogani, Christian Salvatore, Francesca Bertolina, Giuseppe Sarpietro, Mauro Signorelli, Dominique Ronzulli, Isabella Castiglioni, Francesco Raspagliesi

**Affiliations:** 1 Department of Gynecologic Oncology, Fondazione IRCCS Istituto Nazionale dei Tumori di Milano, Via Venezian 1, 20133 Milan, Italy; 2 DeepTrace Technologies S.R.L., Milan, Italy; 3 Clinical Trial Center, Fondazione IRCCS Istituto Nazionale Tumori di Milano, Milan, Italy; 4 Dipartimento di Fisica G. Occhialini, University of Milan-Bicocca, Milan, Italy

**Keywords:** Artificial intelligence, Machine learning, CA-125 antigen, Ovarian neoplasms, Ultrasonography

## Abstract

**Background:**

To evaluate the performance of a decision support system (DSS) based on radiomics and machine learning in predicting the risk of malignancy of ovarian masses (OMs) from transvaginal ultrasonography (TUS) and serum CA-125.

**Methods:**

A total of 274 consecutive patients who underwent TUS (by different examiners and with different ultrasound machines) and surgery, with suspicious OMs and known CA-125 serum level were used to train and test a DSS. The DSS was used to predict the risk of malignancy of these masses (very low *versus* medium-high risk), based on the US appearance (solid, liquid, or mixed) and radiomic features (morphometry and regional texture features) within the masses, on the shadow presence (yes/no), and on the level of serum CA-125. Reproducibility of results among the examiners, and performance accuracy, sensitivity, specificity, and area under the curve were tested in a real-world clinical setting.

**Results:**

The DSS showed a mean 88% accuracy, 99% sensitivity, and 77% specificity for the 239 patients used for training, cross-validation, and testing, and a mean 91% accuracy, 100% sensitivity, and 80% specificity for the 35 patients used for independent testing.

**Conclusions:**

This DSS is a promising tool in women diagnosed with OMs at TUS, allowing to predict the individual risk of malignancy, supporting clinical decision making.

## Key points

- A decision support system (DSS) was developed for predicting malignancy-risk level of ovarian masses.
- The DSS is based on transvaginal ultrasound imaging and serum CA-125 level.
- Radiomics and machine learning techniques were implemented.
- The DSS showed 91% accuracy, 100% sensitivity, and 80% specificity in independent testing.
- The DSS allows predicting individual risk level of ovarian mass malignancy.

## Background

Ovarian cancer (OC) is one of the most lethal cancers in women [1], with over 295,400 cases diagnosed worldwide in 2018 and 184,800 deaths [2]. The lack of accurate screening and diagnostic tools, the scarce symptomatology, abnormal cell metabolism [3], and the rapid spread of disease are the main causes of lethality. Biological markers, such as cancer antigen-125 (CA-125), have been shown highly sensitive but poorly specific for screening, and qualitative transvaginal ultrasonography (TUS) are not informative enough to detect OC at the early stage in the general population [4, 5]. To date, no screening test has proven to reduce ovarian cancer mortality, and ovarian cancer screening is not recommended by any scientific society in the general population. Some societies recommend screening with CA-125 and TUS in high-risk women (*e.g.*, carriers of *BRCA1* and other genes mutations) [6].

The high false positive rate of diagnostic methods leads to often useless surgeries for benign masses at final histology and to the lack of centralisation of oncological cases with worsening of the patient’s prognosis [7]. An adequate and reproducible preoperative diagnosis is therefore of paramount importance [8].

The adnexal ovarian masses appear to have particular characteristics at TUS, helpful in the diagnosis to expert operators. For example, masses with predominantly solid composition, irregular in shape, large, and not presenting underlying shadows are more probably malignant. Based on these characteristics, guidelines support specialised clinicians to classify the risk of cancer of ovarian masses on the bases of only ultrasound features as is for the International Federation of Gynecology and Obstetrics (FIGO) classification [9] or with a combination of ultrasound and biological markers, as is for the “risk of malignancy index” (RMI) [10] or “assessment of different neoplasias in the adnexa” (ADNEX) [11].

These classifications are helpful in high and low risk groups but most of them require the knowledge of a specific terminology [12] and a certain level of experience of the examiner in the assignment of echogenic and structural features to OMs, and are inconclusive for lesions assigned with an intermediate risk.

Radiomics is a quite recent but popular image processing technique applied to radiological medical images, including those obtained with TUS [13–15], based on the extraction of quantitative data about lesion morphology and texture features. These features have been shown to be correlated to the pathophysiology of cancer tissue in a large number of oncological diseases [16] and used in multivariate classification models to discriminate cancerous from non-cancerous tissues [17].

In the case of OC, we have recently developed a classification model based on radiomics applied to ultrasonography to automatically classify lesions with proven benign *versus* malignant histopathology on surgical specimens, allowing a stratification of low *versus* high risk of cancer with a mean 84% accuracy, 79% sensitivity, and 86% specificity [18].

In this work, we evaluated the performance of such classification model when used in combination with CA-125 in predicting the risk level of malignancy of OMs (very low risk, medium-high risk), to be used by specialised clinicians as decision support system (DSS) for a personalised diagnostic and treatment pathway for individual patients. We then tested generalisation and reproducibility of such DSS on a patients’ cohort independent from that used for the development of the previous system, being the DSS used by different examiners with different experience.

## Methods

### Study design and study population

This is a single-centre, observational, retrospective, and prospective clinical study. The study population includes two cohorts.

The first cohort is retrospective and inclusion criteria were as follows: consecutive women with age ≥ 18 years who underwent TUS and were diagnosed with OMs, then scheduled for surgery within 2 weeks after TUS examination at Fondazione IRCCS Istituto Nazionale dei Tumori di Milano from January 1, 2017, to December 31, 2019. CA-125 serum level was required.

The second cohort was prospective and inclusion criteria were as follows: consecutive women with age ≥ 18 years who underwent TUS and were diagnosed with OMs, then scheduled for CA-125 level test and surgery within 2 weeks after US examination at Fondazione IRCCS Istituto Nazionale dei Tumori di Milano from January 1, 2021, to April 31, 2021.

Patients in the prospective cohort and patients still alive and traceable in the retrospective cohort signed the study-specific informed consent. For deceased or untraceable patients, the Principal Investigator has filled in the “replacement form for informed consent” approved by the ethics committee, in compliance with DGPR 679/2016. The study protocol was approved by the Ethical Committee (Protocol MULTIAROMA INT. N157/20 Scientific Responsible Dott. Valentina Chiappa, approved 22/07/2020).

A gynaecologist with more than 10 years of experience (senior experience) performed all US examinations and diagnosis at TUS, saving the 2D image frames in DICOM format with the patient’s ovarian mass acquired in longitudinal and perpendicular sections, respectively. Ultrasound competence was not assessed using quantitative scale considering pass/fail scores (*e.g.*, assessment of ultrasound skills).

For both the cohorts, final histology after surgery was considered as standard reference for the definite diagnosis.

### The DSS

The DSS was based on an ensemble of three radiomic machine learning models, specifically designed to classify the risk level for (i) fully solid OMs (“solid masses”), (ii) fully liquid OMs (“cystic masses”), and (iii) OMs with solid and liquid components (“mixed masses”). Such radiomic machine learning models have been previously proposed and tested on the first patient cohort in [18]. Two hundred and sixty-nine (269) stable radiomics features have been found as stable predictors to characterise the echogenic structure and morphometry of solid OMs by the first model, 278 for the liquid masses by the second model, and 306 for the third one customised for the lesions with mixed solid and liquid components.

An improvement in the design of such radiomic model is considered in this work by integrating the malignancy risk predicted by each of the three TUS radiomic models [18] with the information on the presence/absence of acoustic shadows, and combining the malignancy risk predicted by such TUS-based model with the serum CA-125 level, considering two different thresholds depending from the menopausal status (premenopause/postmenopause as correctors for CA-125 level). Indeed, the presence of acoustic shadows [12] among solid components has been well acknowledged associated with an OM classification of very low risk of malignancy [11]. Thus, we integrated the malignancy risk predicted by the model trained on radiomic features within the ovarian mass with the information on the presence/absence of acoustic shadows near the mass that does not modify such risk (if acoustic shadows are absent) or reduce such risk (if acoustic shadows are present). On the other hand, for patients diagnosed with OMs at TUS, serum CA-125 level higher than of 71 U/mL (double compared to the upper normal level) for postmenopausal women [19] and 200 U/mL for premenopausal women [20, 21], respectively, have been found associated to a classification of medium-high risk of malignancy.

Specifically, according to such a combined risk approach, in this work, we defined the following DSS and predicted risk:

- When an OM is shown by TUS without acoustic shadows and the serum CA-125 level is below the threshold, the DSS predicts the risk level of malignancy based on the classification of the radiomic models (very low risk or medium-high risk);
- When an OM is shown by TUS with acoustic shadows and the serum CA-125 level is below the threshold, the DSS predicts a very low risk of malignancy;
- When an OM is shown by TUS with or without acoustic shadows and the serum CA-125 level is above the threshold, the DSS predicts a medium-high risk of malignancy.

The DSS was implemented in a stand-alone software tool that can be installed on dedicated Microsoft windows workstations, in an on-premise configuration, with the following minimum characteristics: operative system windows 10, processor Intel i5 × 86 64, RAM of 8 GB, and 8 GB of free space on the hard drive.

For each patient’s ovarian mass, the number of TUS images to upload in the software is only one: the 2D image frame saved in DICOM format with the mass acquired in the longitudinal section.

**Fig. 1:**
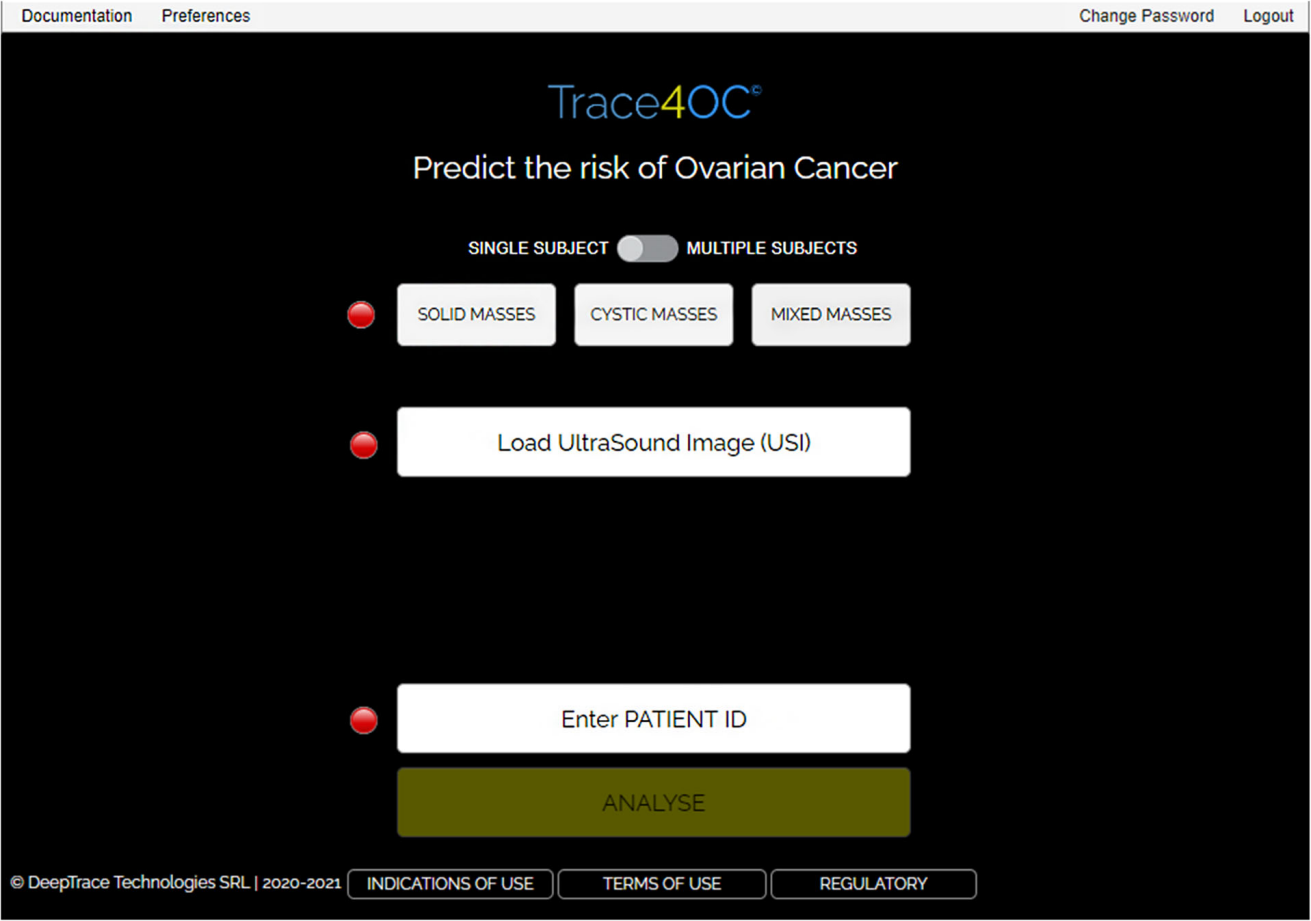
Software interface for the mass types and transvaginal ultrasound image selection

**Fig. 2:**
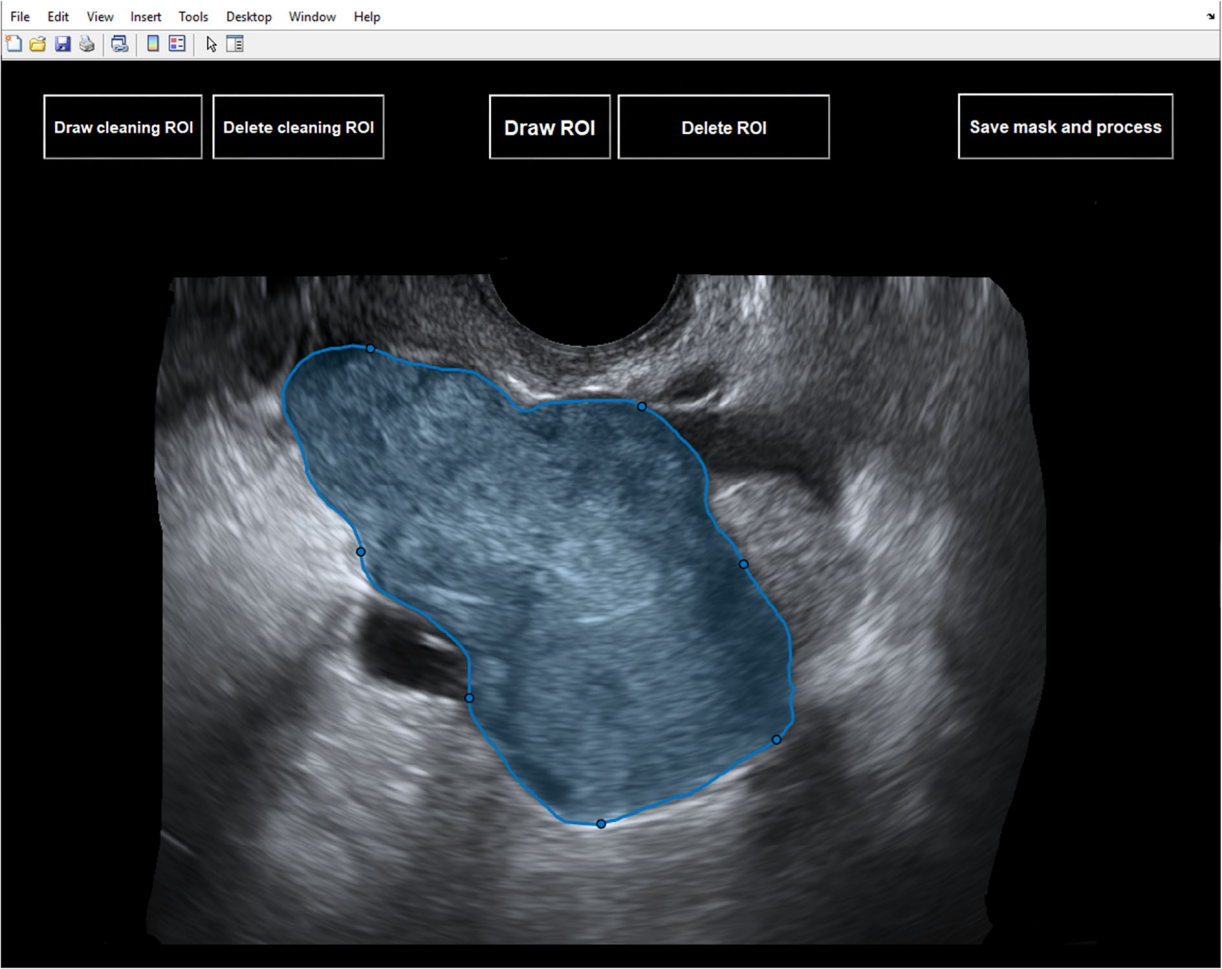
Manual segmentation interface

**Fig. 3:**
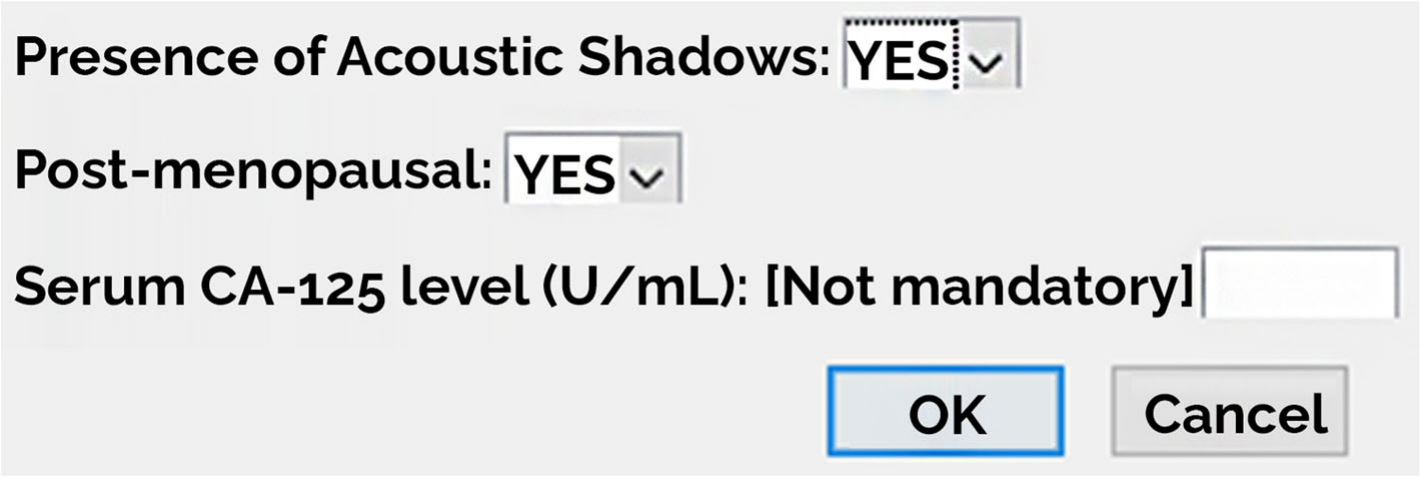
Interface for supplementary information: presence of acoustic shadows, subject menopausal state, and serum CA-125 level

**Fig. 4:**
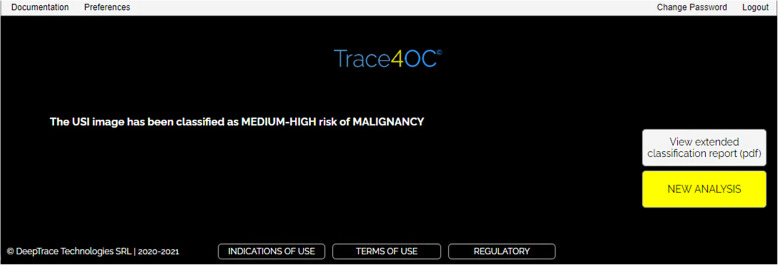
Interface of the classification results

The software tool allows high-resolution viewing of the uploaded TUS image, visual inspection of liquid and solid components of the ovarian mass as well as the presence or absence of acoustic shadow, and fast segmentation of the OMs, through an easy-to-use graphical interface (Fig. 1 and Fig. 2). The segmentation modality is manual, and a process of random manipulations of the contour (small geometrical modifications of the manual contour creating different contours from different operators) is automatically performed by the software to minimise the dependency of the radiomic analysis from the manual segmentation of different operators [18]. Robust radiomic features are automatically calculated and selected by the software for the manipulated segmented mass on the TUS image according to the specific mass type as described in our previous work [18] (269 features for solid masses, 278 features for cystic masses, and 306 features for mixed masses). Such radiomic features are used by one of the three machine learning models, according to the mass type, to predict the risk of malignancy of the mass based only on the TUS radiomic features within the mass. Finally, the supplementary information (*i.e.*, the presence/absence of acoustic shadows at ultrasonography, the menopausal status of the woman, and the serum CA-125 level) is provided to the software (Fig. 3) that predicts the risk of malignancy of the mass (Fig. 4).

In this work, the diagnostic performance and reproducibility of the DSS software was tested by two TUS examiners with different levels of experience: the first one (Examiner 1) was a gynaecologist with less than 2 years of experience (intermediate experience), the second one (Examiner 2) was the gynaecologist with more than 10 years of experience (senior experience).

### Statistical analysis

Data are presented as median and interquartile range (IQR), for patients’ age, frequencies and percentage for mass characteristics, and premenopausal or menopausal status. Diagnostic performances were obtained in terms of sensitivity, specificity, and accuracy comparing the results of the DSS (very low *versus* medium-high) *versus* histopathology reference standard (benign *versus* malignant), with 95% confidence intervals (CI), calculated according to the binomial distribution.

## Results

### Population

From the first cohort, we retrospectively tested 239 women with available TUS images of OMs, serum CA-125 levels, and histopathological results after surgery. TUS images were obtained from a VOLUSON-E8 system (General Electric Healthcare, Chicago, USA). Median age of the patients was 55 years with an IQR of 22 (minimum 18, maximum 84).

Most of patients (61%) referred symptoms before TUS: pelvic or abdominal pain (50%), bloating (20%), increase in abdominal circumference (19%), weight gain or loss (18%), weakness nausea (17%), irregular menstrual periods (14%) (13%), abnormal uterine bleeding (6%), dyspareunia (3%). The characteristics of the three groups of OMs (solid, liquid and mixed) for this first cohort are summarised in Table 1. The majority of patients (62%) were postmenopausal.

**Table 1:** First cohort population

	Number of patients	Mass type	Median age (IQR)	Postmenopausal (%)	Malignant (%)	Malignant, median age (IQR)
First cohort (239 women)	93	Solid	56 (21)	58 (62.4%)	57 (61.3%)	51 (20)
	67	Liquid	51 (22)	36 (53.7%)	12 (17.9%)	54 (19)
	79	Mixed	58 (23)	54 (68.4%)	54 (68.4%)	58 (21)

*IQR* Interquartile range

From the second, independent cohort, we prospectively tested 35 women with available TUS images of OMs, serum CA-125 levels, and histopathological results treated from January 1, 2021, to April 30 2021. TUS images were obtained from a HERA W10 system (Samsung, Seoul, South Korea). Median age of the patients was 50 years with an IQR of 25 (minimum 18, maximum 73). The characteristics of the three groups of OMs (solid, liquid, and mixed) for this second cohort are summarised in Table 2; 57% of patients were postmenopausal.

**Table 2:** Second cohort population

	Number of patients	Mass type	Median age (IQR)	Postmenopausal (%)	Malignant (%)	Malignant, median age (IQR)
Second cohort (35 women)	8	Solid	54 (9)	6 (75.0%)	7 (87.5%)	54 (8)
	13	Liquid	42 (23)	4 (30.8%)	1 (7.7%)	68 (0)
	14	Mixed	54 (29)	10 (71.4%)	12 (85.7%)	58 (23)

*IQR* Interquartile range

Histological characteristics of the OMs after surgery and International Federation of Gynecology and Obstetrics (FIGO) stage in case of malignancy for both cohorts 1 and 2 are summarised in Table 3.

**Table 3:** Histopathological characteristics of ovarian masses included in the study

Variable	Ovarian masses, number (%)
Benign	132 (48.2)
Malignant	142 (51.8)
Total	274 (100.0)
Histopathological type of benign masses	
Serous cystoadenoma/cystoadenofibroma	48 (36.4)
Mucinous cystoadenoma	9 (6.8)
Endometrioma	12 (9.1)
Ovarian fibroma/fibrothecoma	38 (28.8)
Teratoma	8 (6.1)
Pelvic inflammatory disease	2 (1.5)
Peritoneal cyst/pseudocysts	3 (2.3)
Hydrosalpinx	7 (5.3)
Paraovarian cyst / paratubaric cyst	5 (3.8)
Total	132 (100.0)
Histopathological type of malignant masses	
High-grade serous ovarian cancer	58 (40.9)
Low-grade serous ovarian cancer	6 (4.2)
Serous borderline tumour	12 (8.5)
Mucinous borderline tumour gastrointestinal type	7 (4.9)
Mucinous borderline tumour endocervical type	5 (3.5)
Mucinous ovarian cancer	2 (1.4)
Endometrioid ovarian cancer	4 (2.8)
Clear cell ovarian cancer	4 (2.8)
Granulosa cell ovarian tumour	3 (2.1)
Ovarian carcinosarcoma	5 (3.5)
Dysgerminoma	1 (0.7)
Ovarian metastases from other tumour	22 (15.5)
Tubal cancer	1 (0.7)
Yolk sac tumour	3 (2.1)
Immature teratoma	6 (4.2)
Sertoli Leydig tumour	3 (2.1)
Total	142 (100.0)
FIGO stage of primitive ovarian cancers	
IA	26 (21.7)
IB	2 (1.7)
IC1	4 (3.3)
IC2	1 (0.8)
IC3	6 (5.0)
IIA	3 (2.5)
IIB	6 (5.0)
IIIA1	6 (5.0)
IIIA2	1 (0.8)
IIIB	9 (7.5)
IIIC	37 (30.8)
IVA	5 (4.2)
IVB	14 (11.7)
Total	120 (100.0)

*FIGO* International Federation of Gynecology and Obstetrics

### DSS performance

The performance metrics of DSS (specificity, sensitivity, accuracy, positive predictive value, negative predictive value), with 95% CI in square brackets, are shown in Table 4 for the first and second cohorts as well as for the two different examiners using DSS on the same images of the same patients.

**Table 4:** Performances of the decision support system for the two patient cohorts and the two examiners

		Examiner 1	Examiner 2
First cohort (239 women)	Sensitivity	122/123, 99.2% (95.6–100.0)	121/123, 98.4% (94.3–99.8)
	Specificity	88/116, 75.9% (67.0–83.3)	91/116, 78.5% (69.9–85.5)
	Accuracy	210/239, 87.9% (83.0–91.7)	212/239, 88.7% (84.0–92.4)
	PPV	122/150, 81.3% (74.2–87.2)	121/146, 82.9% (75.8–88.6)
	NPV	88/89, 98.9% (93.9–100.0)	91/93, 97.9% (92.5–99.7)
Second cohort (35 women)	Sensitivity	20/20, 100.0% (83.2–100.0)	20/20, 100.0% (83.2–100.0)
	Specificity	12/15, 80.0% (51.9–95.7)	12/15, 80.0% (51.9–95.7)
	Accuracy	32/35, 91.4% (76.9–98.2)	32/35, 91.4% (76.9–98.2)
	PPV	20/23, 86.967% (66.4–97.2)	20/23, 86.967% (66.4–97.2)
	NPV	12/12, 100.0% (74.5–100.0)	12/12, 100.0% (74.5–100.0)

Data are given as ratios, percentages (95% confidence intervals). Note: two of the 35 ovarian masses were defined differently by the two examiners (solid for the first examiner, mixed for the second): however, the decision support system classified them with the same level of risk (medium-high risk)

Serum CA-125 levels above thresholds were found for 81 women (71 and 10 from the first and second cohorts, respectively), corresponding to 30% of the 274 OMs. Acoustic shadows were identified in 46 TUS (37 and 9 from the first and second cohorts, respectively), corresponding to 17% of the 274 OMs. Serum CA-125 levels below threshold was found in 36 of the 46 women with acoustic shadows in their TUS (30 and 6 from the first and second cohorts, respectively), corresponding to 13% of the 274 OMs. The classification of the remaining 157 OMs (138 and 19 from the first and second cohorts, respectively, corresponding to 57% of the 274 OMs) depended on the radiomic models.

**Fig. 5:**
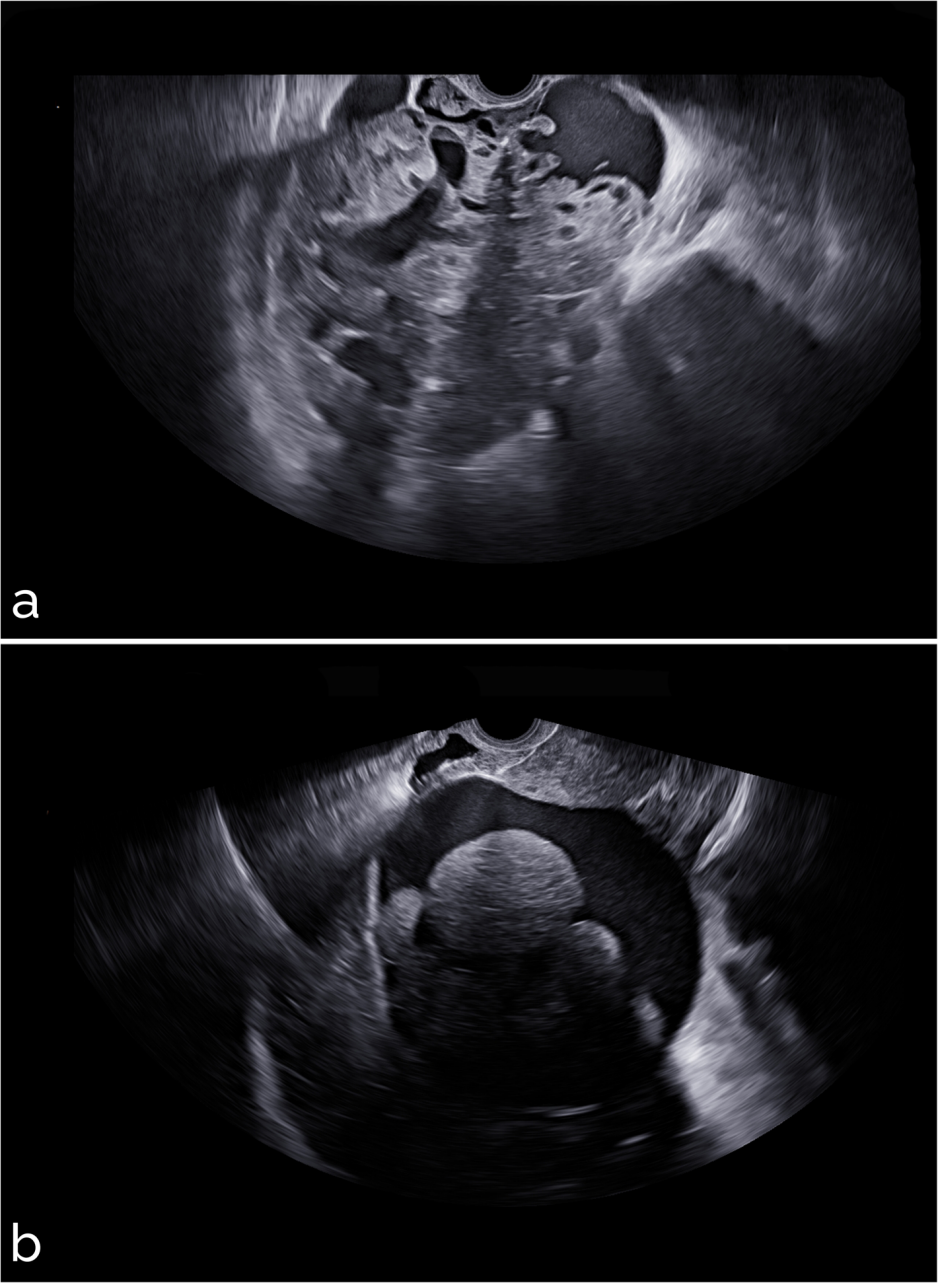
Transvaginal ultrasound of ovarian masses with acoustic shadows: (**a**) solid mass with acoustic shadows, CA125 205 IU/mL, immature teratoma on histological examination (medium-high risk), (**b**) mixed adnexal mass with acoustic shadows, benign teratoma on histological examination (very low risk)

**Fig. 6:**
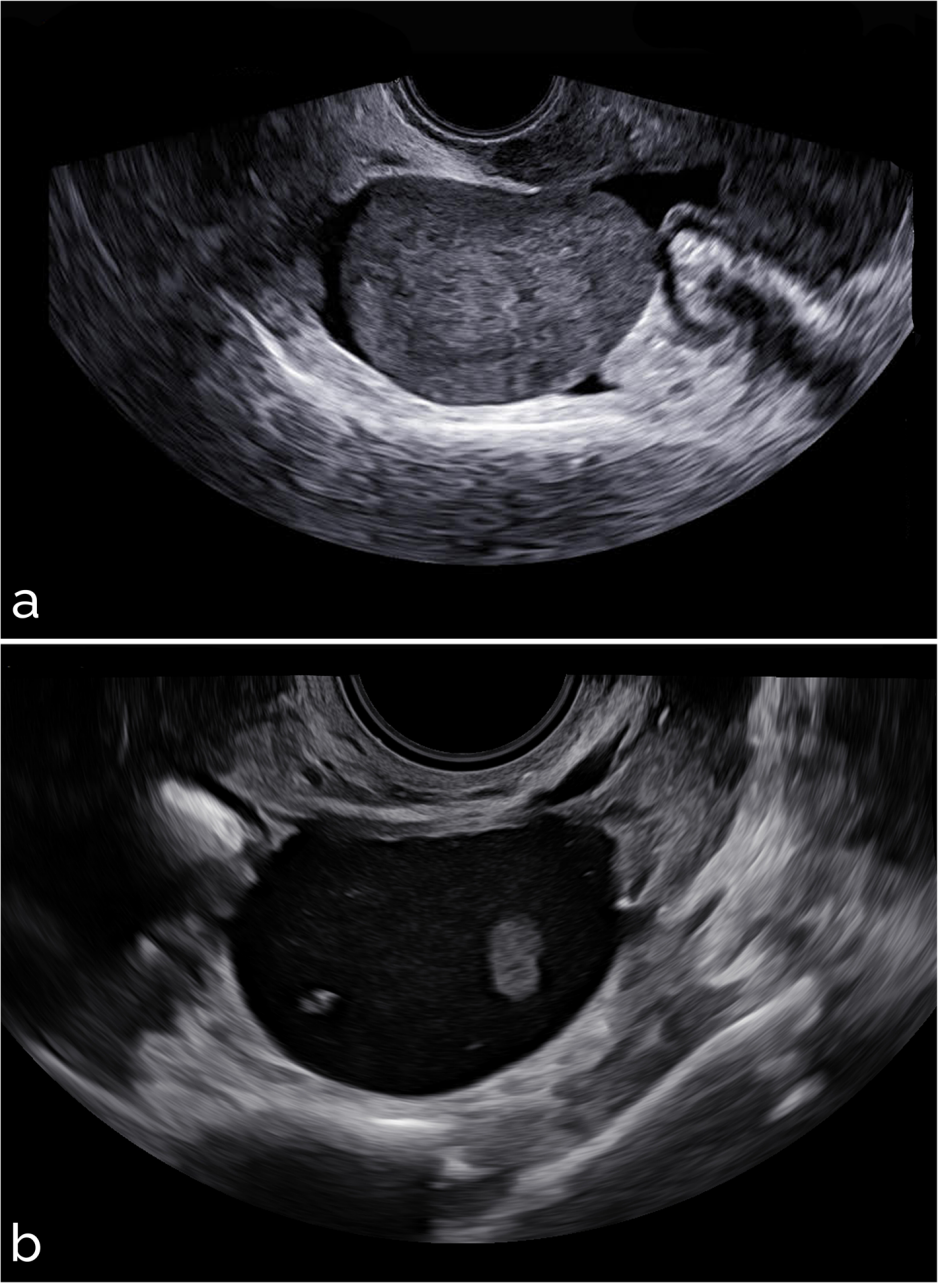
Transvaginal ultrasound of ovarian masses without acoustic shadows: (**a**) solid mass without acoustic shadows, CA 125 12 IU/mL, malignant on histological examination (Krukenberg ovarian tumour) (medium-high risk), (**b**) cystic mass without acoustic shadows, endometrioma at histological examination (very low risk)

**Fig. 7:**
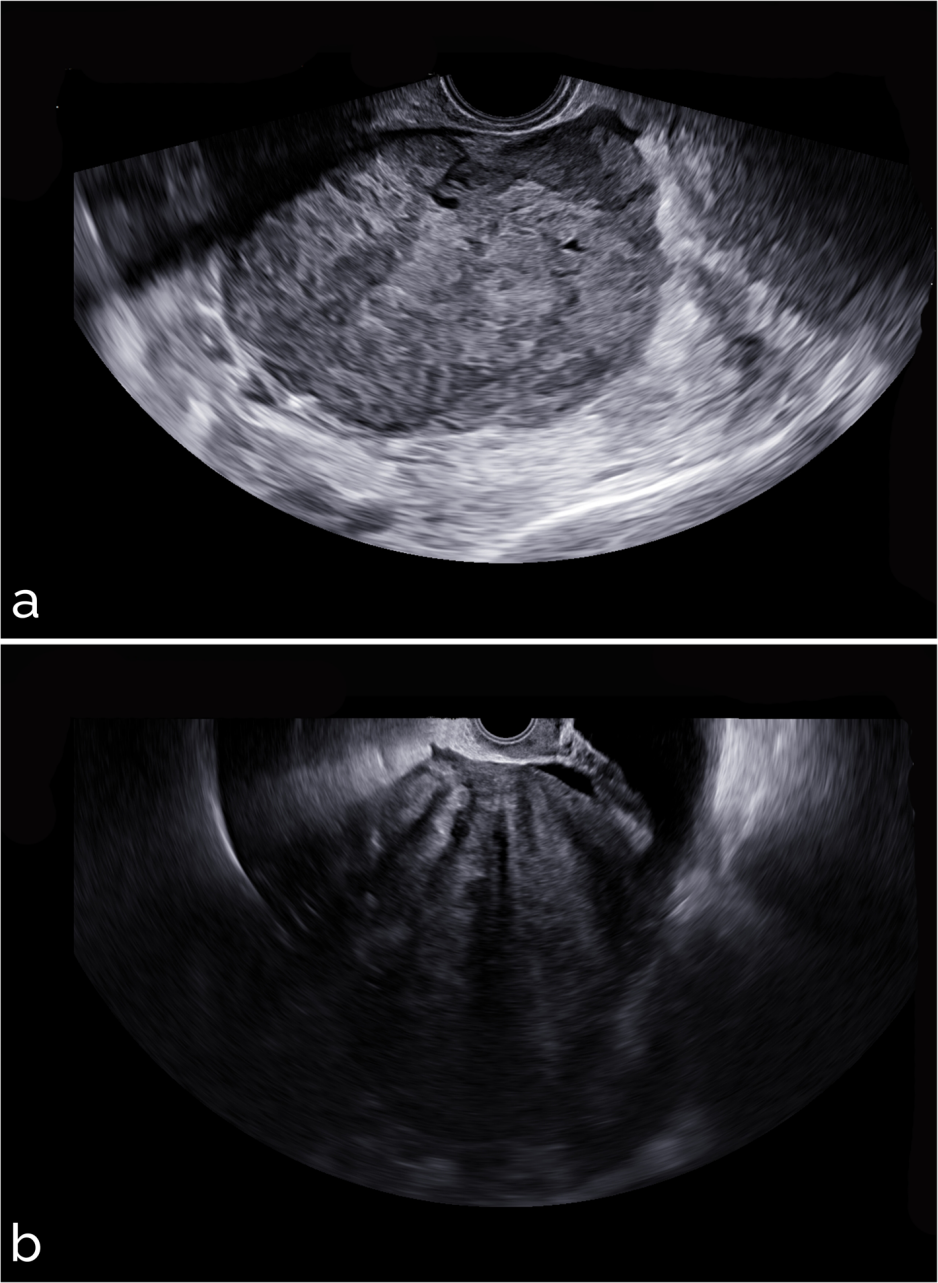
Transvaginal ultrasound of solid ovarian masses: (**a**) clear cell ovarian cancer on histological examination (medium-high risk), (**b**) ovarian fibroma on histological examination (very low risk)

**Fig. 8:**
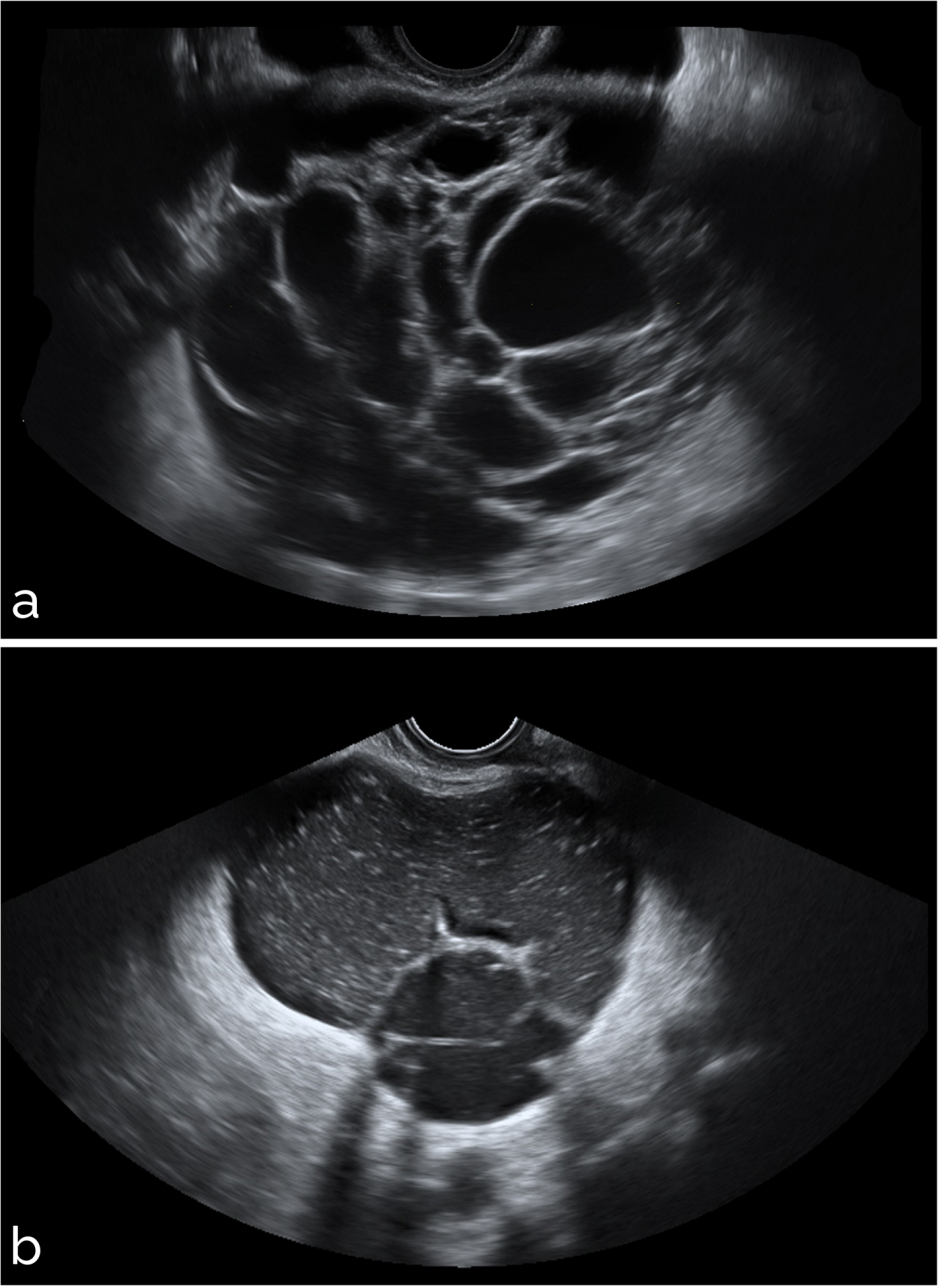
Transvaginal ultrasound of cystic ovarian masses: (**a**) mucinous ovarian cancer on histological examination (medium-high risk), (**b**) mucinous cystoadenoma on histological examination (very low risk)

**Fig. 9:**
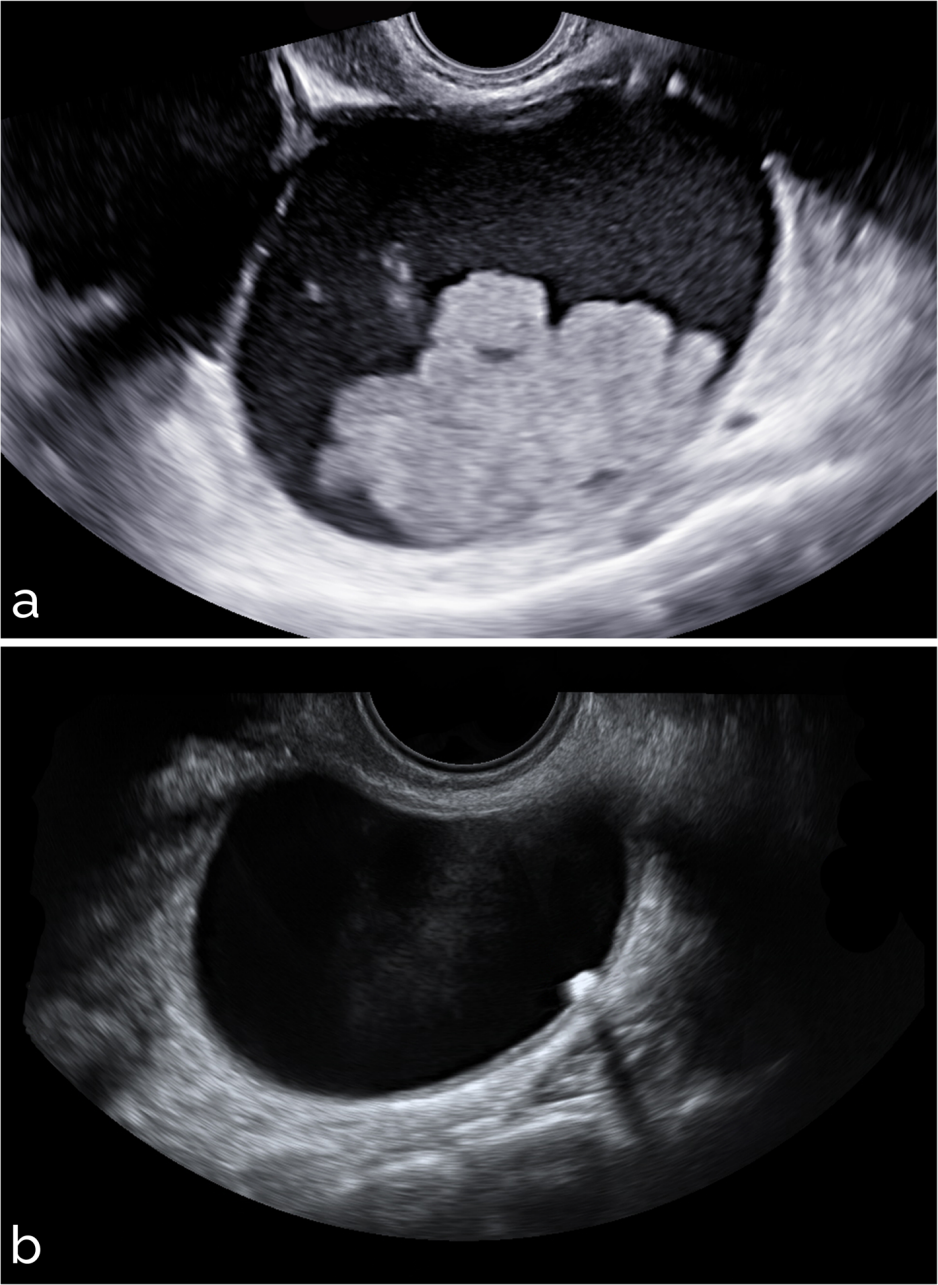
Transvaginal ultrasound of mixed ovarian masses: (**a**) serous borderline tumour on histological examination (medium-high risk), (**b**) cystoadenofibroma on histological examination (very low risk)

Sample images from different settings are shown in Figs. 5, 6, 7, 8, and 9. Specifically, Fig. 5 shows cases with acoustic shadows being classified as medium-high (a) or very low (b) risk. Figure 6 shows cases without acoustic shadows being classified as medium-high (a) or very low (b) risk. Figure 7 shows cases of solid OMs being classified as medium-high (a) or very low (b) risk. Figure 8 shows cases of cystic OMs being classified as medium-high (a) or very low (b) risk. Figure 9 shows cases of mixed OMs being classified as medium-high (a) or very low (b) risk.

## Discussion

AI technology has recently brought an unprecedented growth of applications to medical imaging, and AI predicting models are entering into clinical practice, thanks to the wide availability of digital medical images and the technical advancements in hardware and software architectures compared with the technologies and libraries of the past. AI has been applied to TUS OMs in some studies, albeit not as extensively as some other imaging modalities such as radiography, mammography, computed tomography, and magnetic resonance imaging for solid cancers. An automatic analysis of TUS images based on quantification of gray-level features was proposed by Zimmer et al. [22] obtaining a success rate of 80–90% for benign OMs but only 70% for solid and mixed malignant OMs. An expert system [23] and artificial neural networks [24, 25] were applied to classify US image into benign and malignant but image features were manually measured and provided by the experimenters. Kazendar et al. [26] developed a fully automatic machine learning classifier stratifying US images as benign or malignant masses with an accuracy of 77% when images were enhanced with a Local Binary Pattern operator. An automatic scoring system, HistoScanning, based on the quantification of tissue disorganisation induced by malignant processes in backscattered ultrasound waves before image processing was developed as computer-aided technology able to predict malignant *versus* benign OMs with excellent sensitivity (90%) and high specificity (88%). However, this tool should be embedded in the US system to properly function [27].

In our study, we have created, tested, and prospectively validated a predictive model to triage OMs based on radiomic analysis of TUS images combined with menopausal status and CA125 levels. Our model proved to be an accurate and reproducible DSS for the clinicians with an accuracy of 91% on the validation cohort.

For this purpose, ultrasonography studies from a retrospective cohort of 239 patients and from a prospective cohort of 35 patients were analysed by two different examiners using a stand-alone software tool in which the DSS was implemented, allowing fast manual segmentation of OM, robust radiomic analysis, and classification of risk level of single-subject OMs.

Compared to our first model developed (AROMA pilot study) [18], we have improved the DSS by introducing clinical and biological parameters easy to be obtained (CA-125 value and menopausal status) and an TUS parameter (presence of shadows) that is simple to identify for the examiners because it is a clear, common, and well-known US image feature associated with benign ovarian masses [28].

We have also tested the reproducibility of the tool with different TUS machines and between examiners with different levels of experience with excellent results. Although the subjective impression of the experienced TUS examiner can perform well in defining those OMs to be surgically treated [29], the lack of reproducibility among different examiners represents one of the highest clinical unmet needs in gynaecologic oncology TUS [30]. Our tool aims to bring this gap providing a fair and reproducible approach for assessing the nature of OMs.

A strength of our study is the radiomic analysis according to the IBSI guidelines of the International Biomarker Standardisation Initiative (IBSI), which is a guarantee of feature extraction reproducibility. Moreover, definitive histological examination after surgery—considered as reference standard—was available for all patients.

Furthermore, the model, classifying patients into two classes (very low risk and medium-high risk) overcomes the problem of the “uncertain” OM class: the current recommendations for the “uncertain” class problem are to assess the OMs by second-level imaging (*e.g.*, magnetic resonance imaging) or to address directly to surgery, with a high number of false positives. With our predictive model, the masses in the very low class can be managed conservatively, while the masses in the medium-high risk class can be assessed by second-level imaging (*e.g.*, magnetic resonance imaging) or surgery, with a reduction of about one third in false positives and false negatives. This dichotomy certainly represents an important decision support for less experienced examiners in OMs triage.

The DSS system showed excellent diagnostic performance, not only for the first cohort of patients (retrospective), used to train and test the DSS, but also for the second one (prospective), used as independent test, both in terms of sensitivity and specificity, demonstrating a high generalisation and reproducibility of results with respect to the two different TUS systems and with respect to the two different examiners. This advantage is warranted by random manipulations of manual segmentation of the OMs by an examiner, simulating variations in segmentations miming different examiner segmentations. Radiomic features are selected as stable among these manipulations and this avoids a certain percentage of intrinsic error.

With respect in particular to the examiner reproducibility, it should be noted that, in the first cohort, all OMs were defined in the same class by the two examiners (solid or cystic or mixed), while in the second cohort two of the 35 OMs were defined differently (solid for the first examiner, mixed for the second one): however, the DSS classified them with the same level of risk (medium-high risk). Considering that all the OMs analysed in this study (both for the retrospective and for the prospective court) were sent to the intervention on the basis of the indications of the medical oncologists’ specialists and that the negative predictive value of the DSS is higher than 97%, the tool showed high potential in avoiding treatments to negative patients while maintaining a high ability in selecting the patients to be referred for surgery.

Of note, the predictive values were quite balanced due to the disease prevalence that was 51.5% (123/239) in the first cohort and 57.1% (20/35) in the second independent cohort. This balanced prevalence, as expected [31], enhances the role of the DSS in predicting the risk of malignancy allowing to strongly reduce both false positives and false negatives. We would also specify that all patients enrolled in the study underwent surgery for various reasons: suspicion of malignancy on ultrasound, patient request, symptoms worsening the quality of life, reasons connected with fertility. There were no sources of bias due to the exclusion of very low-risk cases due to human readers.

Our work has some limitations. Compared to the more widely used ADNEX model, our DSS does not stratify the risk of the OM to be a borderline tumour, an early or advanced ovarian cancer or a metastasis [11, 32, 33]. Moreover, the validation will take advantage of cohorts from more centres and larger sample size. All ultrasound examinations were performed by an experienced reader; thus, images may have been leaning towards a higher-than-average quality. We did not perform a classification of ultrasound images by the two human readers into the two risk classes. Given that readers’ experience might be a key issue in both ultrasound image acquisition and interpreting findings from ultrasound concerning ovarian cancer, the comparison on the use of the DSS among users of different experiences including the image acquisition step could possibly highlight the fact that less experienced readers, such as those performing ultrasound examinations in centres with limited ovarian cancer workflows, might benefit from our model to a greater extent or, on the contrary, showing a lower performance when ultrasound images are acquired by less skilled operators. More so, it might be interesting to review whether having a human read parallel to the automatic classification system could possibly lead to an even higher accuracy, leaning towards a double-read system combining imaging features and human insight.

In conclusion, our DSS tool for predicting the risk level of malignancy of OMs through combination of TUS features and clinical/biological parameters shows high accuracy. The tool is semi-automated and provides a reproducible analysis among different TUS machines and examiners that could offer a useful second opinion after an only qualitative visual analysis of TUS images by a single examiner, supporting the clinical decision making. The results obtained in the “real-world” through prospective validation on the considered cohort of patients are exciting and will be monitored on other cohorts in a multicentre setting.

## Data Availability

The datasets used and/or analysed during the current study are available from the corresponding author on reasonable request.

## Abbreviations

ADNEX: Assessment of different neoplasias in the adnexa;
CA-125: Cancer antigen-125
CI: Confidence interval
DICOM: Digital imaging and communications in medicine
DSS: Decision support system
FIGO: International Federation of Gynecology and Obstetrics
IBSI: International biomarker standardisation initiative
IQR: Interquartile range
OC: Ovarian cancer
OM: Ovarian mass
TUS: Transvaginal ultrasonography

## References

[1] Ferlay J, Colombet M, Soerjomataram I, et al (2021) Cancer statistics for the year 2020: An overview. Int J Cancer. 10.1002/ijc.33588 10.1002/ijc.33588 33818764

[2] Ferlay J, Colombet M, Soerjomataram I (2019). Estimating the global cancer incidence and mortality in 2018: GLOBOCAN sources and methods. Int J Cancer.

[3] Podo F, Sardanelli F, Iorio E (2007). Abnormal choline phospholipid metabolism in breast and ovary cancer: molecular bases for noninvasive imaging approaches. Curr Med Imaging Rev.

[4] Menon U, Gentry-Maharaj A, Hallett R (2009). Sensitivity and specificity of multimodal and ultrasound screening for ovarian cancer, and stage distribution of detected cancers: results of the prevalence screen of the UK Collaborative Trial of Ovarian Cancer Screening (UKCTOCS). Lancet Oncol.

[5] Pinsky PF, Yu K, Kramer BS (2016). Extended mortality results for ovarian cancer screening in the PLCO trial with median 15 years follow-up. Gynecol Oncol.

[6] Lowry KP, Lee SI (2017). Imaging and screening of ovarian cancer. Radiol Clin N Am.

[7] Woo YL, Kyrgiou M, Bryant A, Everett T, Dickinson HO (2012). Centralisation of services for gynaecological cancers - a Cochrane systematic review. Gynecol Oncol.

[8] Miller RW, Ueland FR (2012). Risk of malignancy in sonographically confirmed ovarian tumors. Clin Obstet Gynecol.

[9] Prat J, FIGO Committee on Gynecologic Oncology (2014). Staging classification for cancer of the ovary, fallopian tube, and peritoneum. Int J Gynaecol Obstet.

[10] Jacobs I, Oram D, Fairbanks J, Turner J, Frost C, Grudzinskas JG (1990). A risk of malignancy index incorporating CA 125, ultrasound and menopausal status for the accurate preoperative diagnosis of ovarian cancer. Br J Obstet Gynaecol.

[11] Van Calster B, Van Hoorde K, Valentin L (2014). Evaluating the risk of ovarian cancer before surgery using the ADNEX model to differentiate between benign, borderline, early and advanced stage invasive, and secondary metastatic tumours: prospective multicentre diagnostic study. BMJ.

[12] Timmerman D, Valentin L, Bourne TH (2000). Terms, definitions and measurements to describe the sonographic features of adnexal tumors: a consensus opinion from the International Ovarian Tumor Analysis (IOTA) Group. Ultrasound Obstet Gynecol.

[13] Nougaret S, Tardieu M, Vargas HA (2019). Ovarian cancer: an update on imaging in the era of radiomics. Diagn Interv Imaging.

[14] Kumbhare D, Shaw S, Ahmed S, Noseworthy MD (2020). Quantitative ultrasound of trapezius muscle involvement in myofascial pain: comparison of clinical and healthy population using texture analysis. J Ultrasound.

[15] Chiappa V, Interlenghi M, Salvatore C (2021). Using rADioMIcs and machine learning with ultrasonography for the differential diagnosis of myometRiAL tumors (the ADMIRAL pilot study). Radiomics and differential diagnosis of myometrial tumors. Gynecol Oncol.

[16] Yeh AC, Li H, Zhu Y (2019). Radiogenomics of breast cancer using dynamic contrast enhanced MRI and gene expression profiling. Cancer Imaging.

[17] Aerts HJ, Velazquez ER, Leijenaar RT (2014). Decoding tumour phenotype by noninvasive imaging using a quantitative radiomics approach. Nat Commun.

[18] Chiappa V, Bogani G, Interlenghi M, et al (2020) The Adoption of Radiomics and machine learning improves the diagnostic processes of women with Ovarian MAsses (the AROMA pilot study). J Ultrasound 10.1007/s40477-020-00503-5 10.1007/s40477-020-00503-5 PMC8572235 32696414

[19] Karlsen MA, Sandhu N, Høgdall C (2012). Evaluation of HE4, CA125, risk of ovarian malignancy algorithm (ROMA) and risk of malignancy index (RMI) as diagnostic tools of epithelial ovarian cancer in patients with a pelvic mass. Gynecol Oncol.

[20] ACOG Committee on Gynecologic Practice (2003). The role of the generalist obstetrician-gynecologist in the early detection of ovarian cancer. Int J Gynaecol Obstet.

[21] ACOG Committee on Practice Bulletins—Gynecology (2016). Practice Bulletin No. 174: Evaluation and management of adnexal masses. Obstet Gynecol.

[22] Zimmer Y, Tepper R, Akselrod S (2003). An automatic approach for morphological analysis and malignancy evaluation of ovarian masses using B-scans. Ultrasound Med Biol.

[23] Bruning J, Becker R, Entezami M (1997). Knowledge-based system ADNEXPERT to assist the sonographic diagnosis of adnexal tumors. Methods Inf Med.

[24] Tailor A, Jurkovic D, Bourne TH, Collins WP, Campbell S (1999). Sonographic prediction of malignancy in adnexal masses using an artificial neural network. Br J Obstet Gynaecol.

[25] Biagiotti R, Desii C, Vanzi E, Gacci G (1999). Predicting ovarian malignancy: application of artificial neural networks to transvaginal and color Doppler flow US. Radiology.

[26] Khazendar S, Sayasneh A, Al-Assam H (2015). Automated characterisation of ultrasound images of ovarian tumours: the diagnostic accuracy of a support vector machine and image processing with a local binary pattern operator. Facts Views Vis Obgyn.

[27] Lucidarme O, Akakpo JP, Granberg S (2010). A new computer-aided diagnostic tool for non-invasive characterisation of malignant ovarian masses: results of a multicentre validation study. Eur Radiol.

[28] Sladkevicius P, Valentin L (2014). Interobserver agreement in describing the ultrasound appearance of adnexal masses and in calculating the risk of malignancy using logistic regression models. Clin Cancer Res.

[29] Timmerman D (2004). The use of mathematical models to evaluate pelvic masses; can they beat an expert operator?. Best Pract Res Clin Obstet Gynaecol.

[30] Coelho Neto MA, Roncato P, Nastri CO, Martins WP (2015). True Reproducibility of UltraSound Techniques (TRUST): systematic review of reliability studies in obstetrics and gynecology. Ultrasound Obstet Gynecol.

[31] Zhang Z, Bullock RG, Fritsche H (2019). Adnexal mass risk assessment: a multivariate index assay for malignancy risk stratification. Future Oncol.

[32] Van Calster B, Van Hoorde K, Froyman W (2015). Practical guidance for applying the ADNEX model from the IOTA group to discriminate between different subtypes of adnexal tumors. Facts Views Vis Obgyn.

[33] Van Calster B (2017). External validation of ADNEX model for diagnosing ovarian cancer: evaluating performance of differentiation between tumor subgroups. Ultrasound Obstet Gynecol.

